# Structure and mechanism of a dehydratase/decarboxylase enzyme couple involved in polyketide β-methyl branch incorporation

**DOI:** 10.1038/s41598-020-71850-w

**Published:** 2020-09-18

**Authors:** Asha V. Nair, Alice Robson, Thomas D. Ackrill, Marisa Till, Matthew J. Byrne, Catherine R. Back, Kavita Tiwari, Jonathan A. Davies, Christine L. Willis, Paul R. Race

**Affiliations:** 1 School of Biochemistry, University of Bristol, University Walk, Bristol, BS8 1TD UK; 2 School of Chemistry, University of Bristol, Cantock’s Close, Bristol, BS8 1TS UK; 3 BrisSynBio Synthetic Biology Research Centre, University of Bristol, Life Sciences Building, Tyndall Avenue, Bristol, BS8 1TQ UK

**Keywords:** Biocatalysis, Biosynthesis

## Abstract

Complex polyketides of bacterial origin are biosynthesised by giant assembly-line like megaenzymes of the type 1 modular polyketide synthase (PKS) class. The *trans*-AT family of modular PKSs, whose biosynthetic frameworks diverge significantly from those of the archetypal *cis*-AT type systems represent a new paradigm in natural product enzymology. One of the most distinctive enzymatic features common to *trans*-AT PKSs is their ability to introduce methyl groups at positions β to the thiol ester in the growing polyketide chain. This activity is achieved through the action of a five protein HCS cassette, comprising a ketosynthase, a 3-hydroxy-3-methylglutaryl-CoA synthase, a dehydratase, a decarboxylase and a dedicated acyl carrier protein. Here we report a molecular level description, achieved using a combination of X-ray crystallography, in vitro enzyme assays and site-directed mutagenesis, of the bacillaene synthase dehydratase/decarboxylase enzyme couple PksH/PksI, responsible for the final two steps in β-methyl branch installation in this *trans*-AT PKS. Our work provides detailed mechanistic insight into this biosynthetic peculiarity and establishes a molecular framework for HCS cassette enzyme exploitation and manipulation, which has future potential value in guiding efforts in the targeted synthesis of functionally optimised ‘non-natural’ natural products.

## Introduction

Polyketides are a large family of structurally complex and functionally diverse bioactive natural products. These molecules frequently exhibit antibiotic, anticancer, antiparasitic, or antifungal activities, and as such have found widespread application as pharmaceuticals and agrochemicals^1–3^. In bacteria, the biosynthetic machinery responsible for the assembly of polyketides often takes the form of large multi-enzyme complexes of the type 1 modular polyketide synthase (PKS) class. These megasynthases possess a distinctive assembly-line like architecture comprised of linearly arranged multi-domain extension modules, housed in sequence within giant polypeptide chains^4,5^. Biosynthesis upon these systems proceeds in a stepwise fashion, via the sequential addition of simple acyl extender units, derived from their parent Coenzyme A (CoA) thioesters, to the growing product chain^6^. Each module within the synthase is minimally responsible for a single chain extension event, following which the nascent product chain is transferred to a neighbouring down-stream module for further processing^7^. This biosynthetic logic provides an expedient route to the assembly of natural product scaffolds of considerable size and complexity, constructed using simple precursor derived substrates, in a manner intimately dependent on synthase architecture.

During biosynthesis following addition of each acyl monomer, the resultant β-keto thiol ester may be tailored by module embedded *cis*-acting enzymatic domains. These modifications include reduction of the ketone catalysed by ketoreductases (KR), followed by dehydration using dehydratases (DH) and a further reduction by enoyl reductases (ER), or the introduction of α-methyl substituents by C-methyltransferase (CMeT) domains, which employ *S*-adenosyl methionine as a methyl source^8^. These modifications diversify polyketide chemistry and are frequently essential for the bioactivity of the final product^9^. In addition, modifications may be catalysed by free-standing *trans*-acting enzymes, which access and selectively modify intermediates during biosynthesis. Unlike module embedded *cis*-acting domains, *trans*-acting enzymes selectively interact with the synthase complex at designated locations, performing site-specific chemical transformations^10^. Examples include the attachment of acyl extender units onto carrier proteins, or the reductive modification of polyketide intermediates. Systems rich in *trans*-acting elements, as typified by the *trans*-AT family of modular PKSs, contravene many of the established collinearity rules of the well-studied *cis*-AT class of synthases, and consequently represent a rich source of novel natural product chemistry^11,12^.

One distinctive example of *trans*-acting PKS enzymology, which is common though not exclusive to *trans*-AT synthases, is the incorporation of methyl branches at positions β to the thiol ester in the growing polyketide chain. The biosynthetic machinery responsible for this process takes the form of a five-protein HCS cassette comprising a ketosynthase (KS), a 3-hydroxy-3-methylglutaryl-CoA synthase (HCS-CoA), a dehydratase, a decarboxylase and a stand-alone acyl carrier protein (ACP)^13,14^. The chemical logic employed during HCS-cassette dependent β-methyl branching is analogous to that observed in isoprenoid biosynthesis^15,16^. Branch formation generally occurs upon distinctive di-ACP, or less commonly tri-ACP domains, present within the core PKS assembly line, with branching enzymes recruited to these sites by signature amino acid motifs^17^. The specificity of these protein–protein interactions affords a degree of control over branch placement, opening up intriguing possibilities for the relocation of synthase embedded ACPs upon which branches are assembled to alternative sites within HCS-cassette containing PKSs. An alternative approach is the transfer of HCS-cassette enzymes and their cognate ACP domains into heterologous systems, in an effort to direct targeted branch incorporation into non-branched polyketides^18^.

**Figure 1:**
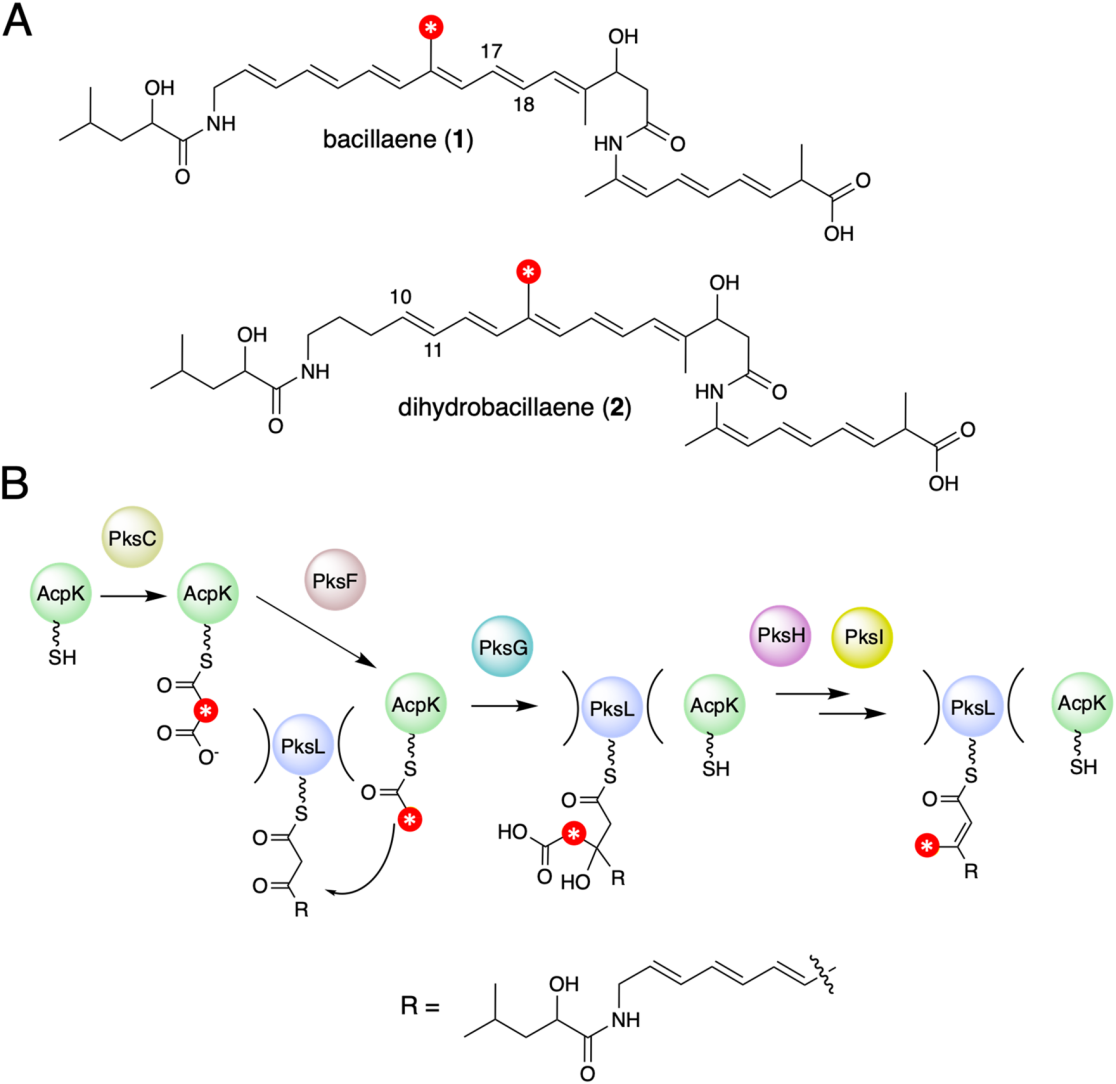
β-Methyl branch incorporation during biosynthesis of the bacillaenes. (**A**) Chemical structures of bacillaene stereoisomer (**1**) and dihydrobacillane stereoisomer (**2**). Reported isomers include the C17–C18 *trans* double-bond isomer of **1** and the C10–C11 *trans* double-bond isomer of **2**^19^. (**B**) Mechanism of HCS cassette dependent β-branching in bacillaene biosynthesis.

The bacillaenes (**1**) and their analogues the dihydrobacillaenes (**2**), are polyene diamide polyketide/non-ribosomal peptides, produced as mixtures of stereoisomers (Fig. 1A)^19^. The bacillaene synthase was one of the first *trans*-AT systems to be identified and it has subsequently served as a useful model system for establishing many of the general principles of *trans*-AT PKS enzymology^20–22^. The hybrid polyketide synthase/non-ribosomal peptide synthetase (PKS/NRPS) responsible for the biosynthesis of these compounds is encoded for by an ~ 80 kb gene cluster found on the genomes of *Bacillus subtilis* 168 and *Bacillus amyloliquefaciens* FZB42^23,24^. Bacillaene is an effective inhibitor of prokaryotic protein synthesis, though its chemical instability is likely to preclude its development as a clinically useful antibiotic^25^. Bacillaene contains a single β-methyl branch within the conjugated hexene moiety of the molecule, which is installed on the di-ACP domain of the of the multimodular protein PksL. Overall addition of the β-methyl group is catalyzed by the HCS cassette enzymes PksF (ketosynthase), PksG (HCS), PksH (decarboxylase) and PksI (dehydratase), with acylated AcpK, a free-standing acyl carrier protein encoded within the HCS cluster, providing the nucleophile for attack onto the β-keto thioester on PksL (Fig. 1B)^13^. Although β-branching enzymes from PKSs have been subjected to some structural and functional characterization, much still remains to be learned about the enzymology of HCS cassette polypeptides^26–29^. In light of this, we describe herein crystal structures of the bacillaene synthase β-branching enzymes PksH and PksI, which are shown to adopt related trimeric crotonase superfamily (CS) folds. Structure guided mutagenesis in tandem with enzyme assays using synthesized substrates, enables the identification of key catalytic residues in both enzymes and the elucidation of their reaction mechanisms. These data represent the first mechanistic description of a HCS cassette dehydratase, and establish a deviant catalytic mechanism in PksI, which diverges from that previously proposed in other HCS cassette decarboxylases^29^. Despite both enzymes adopting analogous folds and oligomeric states, we could find no evidence for the formation of heterotrimeric complexes when PksH and PksI were co-expressed in *Escherichia coli*, implying fidelity in the folding and assembly of the trimeric functional forms of these proteins. Together these data provide a comprehensive molecular description of the terminal steps in β-methyl branching in bacillaene biosynthesis, findings that are directly applicable to equivalent processes in both *cis*- and *trans*-AT PKSs.

## Results

### Crystal structure of PksH

**Figure 2:**
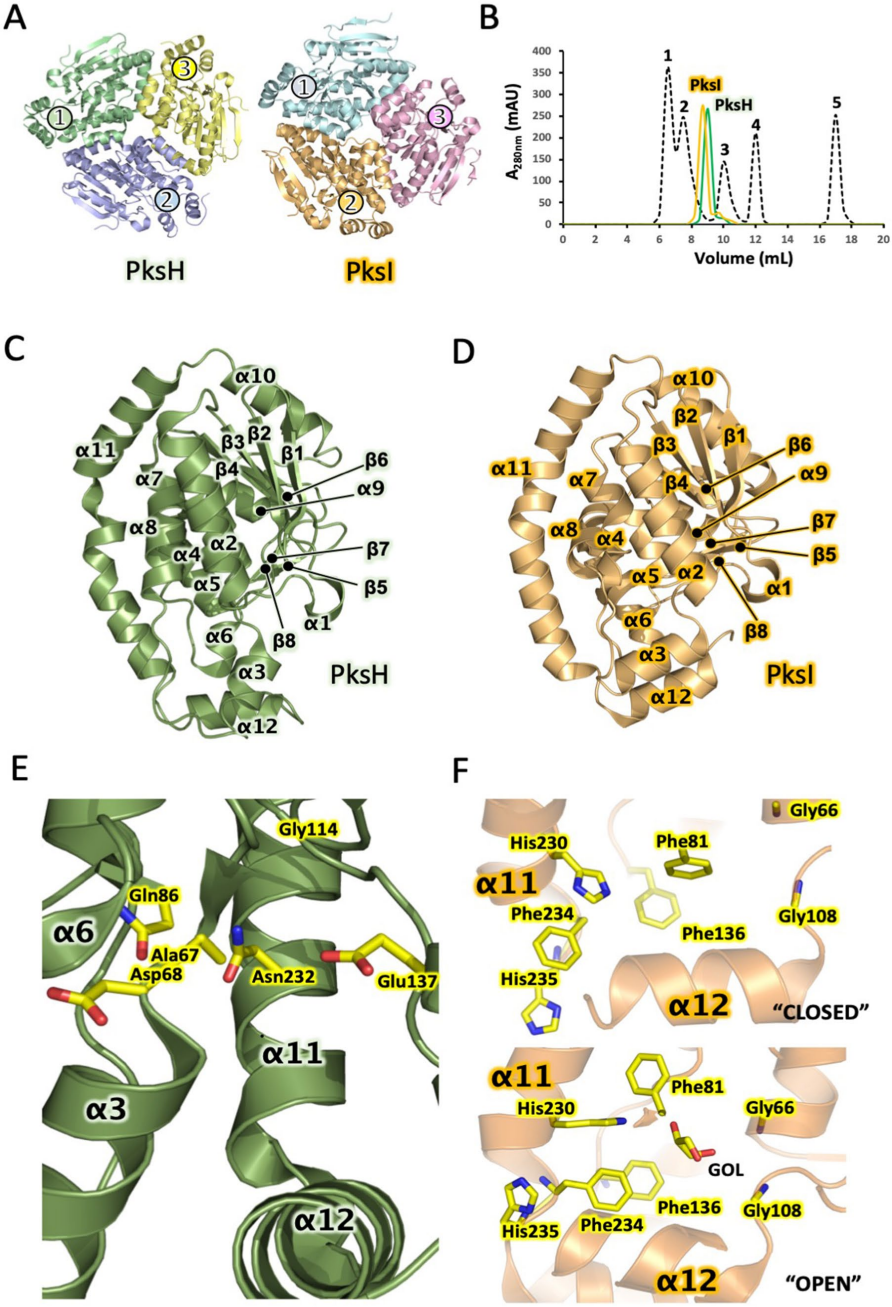
Structural studies of PksH and PksI. (**A**) X-ray crystal structures of PksH and PksI. Individual monomers comprising the PksH and PksI trimers are differentially coloured with colour match circles indicating the location of individual active sites within each monomer. (**B**) Chromatogram showing the elution profiles of PksH (green) and PksI (orange) from a Superdex 200 10/60 column (GE Healthcare) pre-equilibrated in 20 mM Tris–HCl, 150 mM NaCl, pH 7.5. 0.5 mg of each protein was loaded onto the column. Standards, in order of elution, are 1. thyroglobulin Ve = 10.1 (Mr = 670,000 Da), 2. γ-globulin Ve = 13.3 (Mr = 158,000 Da), 3. ovalbumin Ve = 15.2 (Mr = 44,000 Da), 4. myoglobin Ve = 16.8 (Mr = 17,000 Da) and 5. vitamin B12 Ve = 19.6 (Mr = 1,350 Da). (**C**) X-ray crystal structure of a single PksH monomer. (**D**) X-ray crystal structure of a single PksI monomer. (**E**) Details of the PksH active site with key amino acids and secondary structure elements highlighted. (**F**) Details of the PksI active site in both “closed” and “open” states, with key amino acids and secondary structure elements highlighted.

The crystal structure of the dehydratase PksH (PDB 3HP0) has been determined to 2.3 Å resolution. Although deposited in the PDB, this structure is yet to be published. In light of this, we first undertook a detailed analysis of the PksH structure in an effort to establish a putative reaction mechanism for this enzyme. The final model of PksH describes an asymmetric unit (AU) comprised of six protein chains. There are no significant structural differences between each of the six chains that form the AU, with a maximum Cα r.m.s.d between individual chains of 0.48 Å. PksH is trimeric (Fig. 2A), with dimensions of 66 Å × 75 Å × 32 Å. Each trimer is formed across a crystal symmetry axis. A total of 17% (1,207 Å^2^) of the solvent-accessible surface area of each PksH monomer is buried at the trimer interface indicative that this oligomeric state is obligatory. As would be expected, the buried trimer interface is dominated by hydrophobic amino acids. The oligomeric state of PksH observed in the crystal structure is consistent with our hydrodynamic analyses that suggest that this enzyme is trimeric both in solution and *in crystallo* (Fig. 2B).

Each monomer within the PksH trimer possesses a single active site and these are positioned equidistant ~ 57 Å apart within the trimer. Each active site of PksH sits at the base of a solvent exposed channel positioned on the surface of the molecule (Fig. 2C). The fold of PksH is consistent with other CS members, with a characteristic ββα fold, consisting of two perpendicular β-sheets flanked by α-helices^30^. PksH contains a total of 8 β-strands (β1–β8), organized into a pair of β-sheets. The first of these is formed by strands β1, β2, β3, β5 and β7, the second by β4, β6, and β8 (Fig. 2C).

### Active architecture of site of PksH

Despite sharing < 20% amino acid sequence identity with other structurally characterized CS members, PksH retains the distinctive protein fold common to these polypeptides. Superposition of the structure of PksH with those of related homologues thus enabled the unambiguous identification of the active site of the enzyme, along with a number of critical elements therein. These include the active site access tunnel, a putative oxyanion hole, and to a lesser extent, tentative assignment of catalytic residues within the enzyme active site. The identification of these critical features is complicated by the highly diverse sequences of CS proteins. This translates into divergent amino acid composition within the active sites of different superfamily members. Despite this, structural alignment of PksH with other CS polypeptides clearly indicates that the backbone amides of residues Ala67 and Gly114 form the oxyanion hole in this enzyme (Fig. 2E), which in the PksH structure is occupied by a single water molecule. The active site is comprised of the residues Asp68, Asn232, Gln86, Glu137, Ala67 and Gly114, of which Asp68 and Glu137 appear to be optimally positioned to contact the 3-hydroxy-3-methylglutaryl group of the authentic HMG-ACP substrate. This is based on both their respective locations within the enzyme active site and their proximity to the oxyanion hole.

### Enzyme assays and catalytic mechanism of PksH

**Figure 3:**
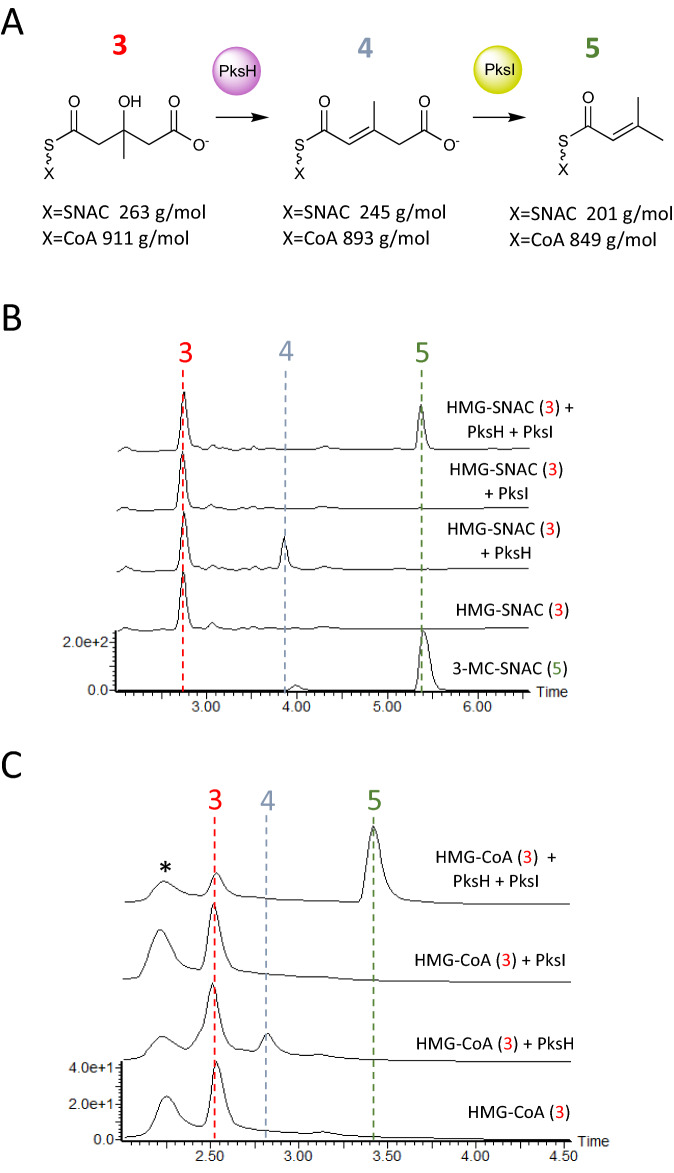
PksH and PksI enzyme activity assays. (**A**) Reaction scheme for the PksH/PksI conversion of substrate analogues (**3**) to products (**4**) and (**5**). Free acid molecular weights of the SNAC and CoA derivatives of the compounds are shown below each structure. (**B**) LCMS diode-array detector chromatograms (210–400 nm) of assays for PksH and PksI using HMG-SNAC substrates. (**C**) LCMS diode-array detector chromatograms (210–400 nm) of assays for PksH and PksI using HMG-CoA. Peaks correspond to HMG (**3**), 3-MG (**4**) and 3-MC (**5**) derivatives. The peak marked * contained free CoA. The chromatogram for synthetic 3-MC SNAC (**5**) is also shown in (**B**). Corresponding mass spectra are shown in SI.

**Figure 4:**
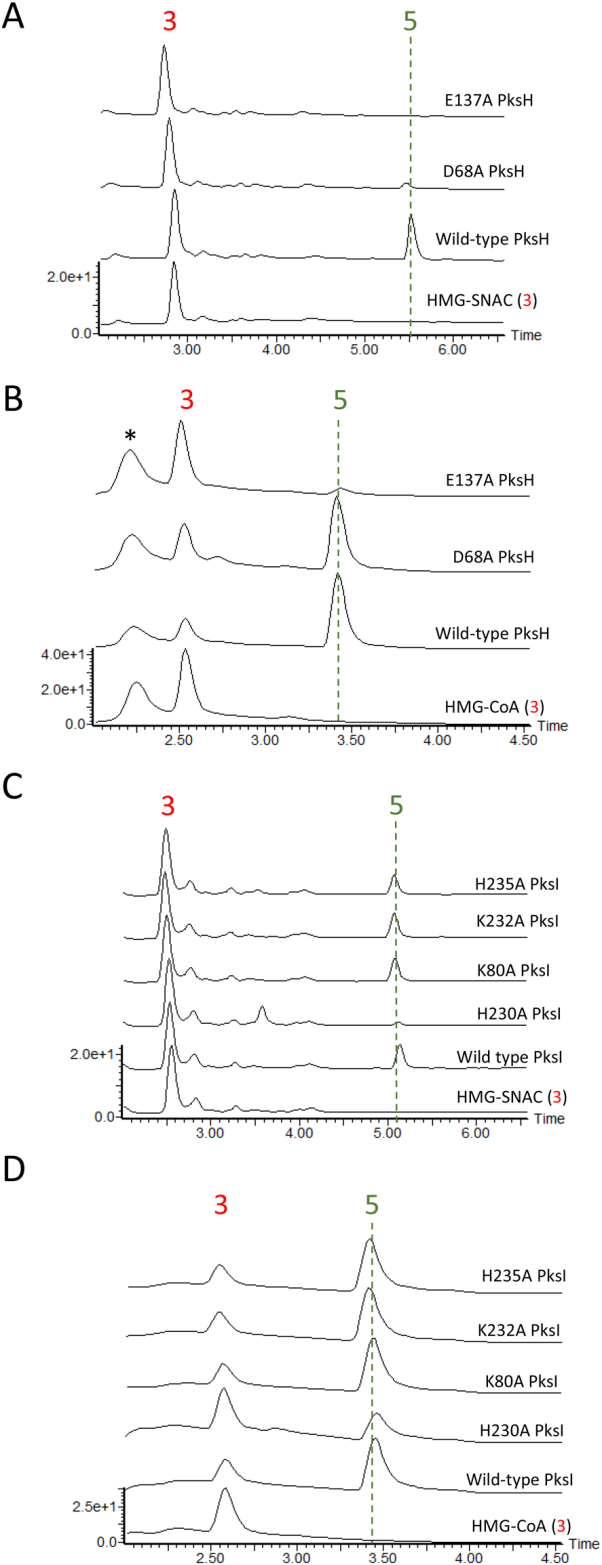
Enzyme activity assays using PksH and PksI mutants. (**A**) LCMS chromatograms for PksH/PksI coupled assays incorporating mutants of PksH and WT PksI using the substrate HMG-SNAC. (**B**) LCMS chromatograms for PksH/PksI coupled assays incorporating WT PksH and mutants of PksI using the substrate HMG-SNAC. (**C**) LCMS chromatograms for PksH/PksI coupled assays incorporating mutants of PksH and WT PksI using the substrate HMG-CoA. (**D**) LCMS chromatograms for PksH/PksI coupled assays incorporating WT PksH and mutants of PksI using the substrate HMG-CoA. All chromatograms were recorded in the 210–400 nm range using a diode-array detector. The peak marked * contained free CoA.

**Figure 5:**
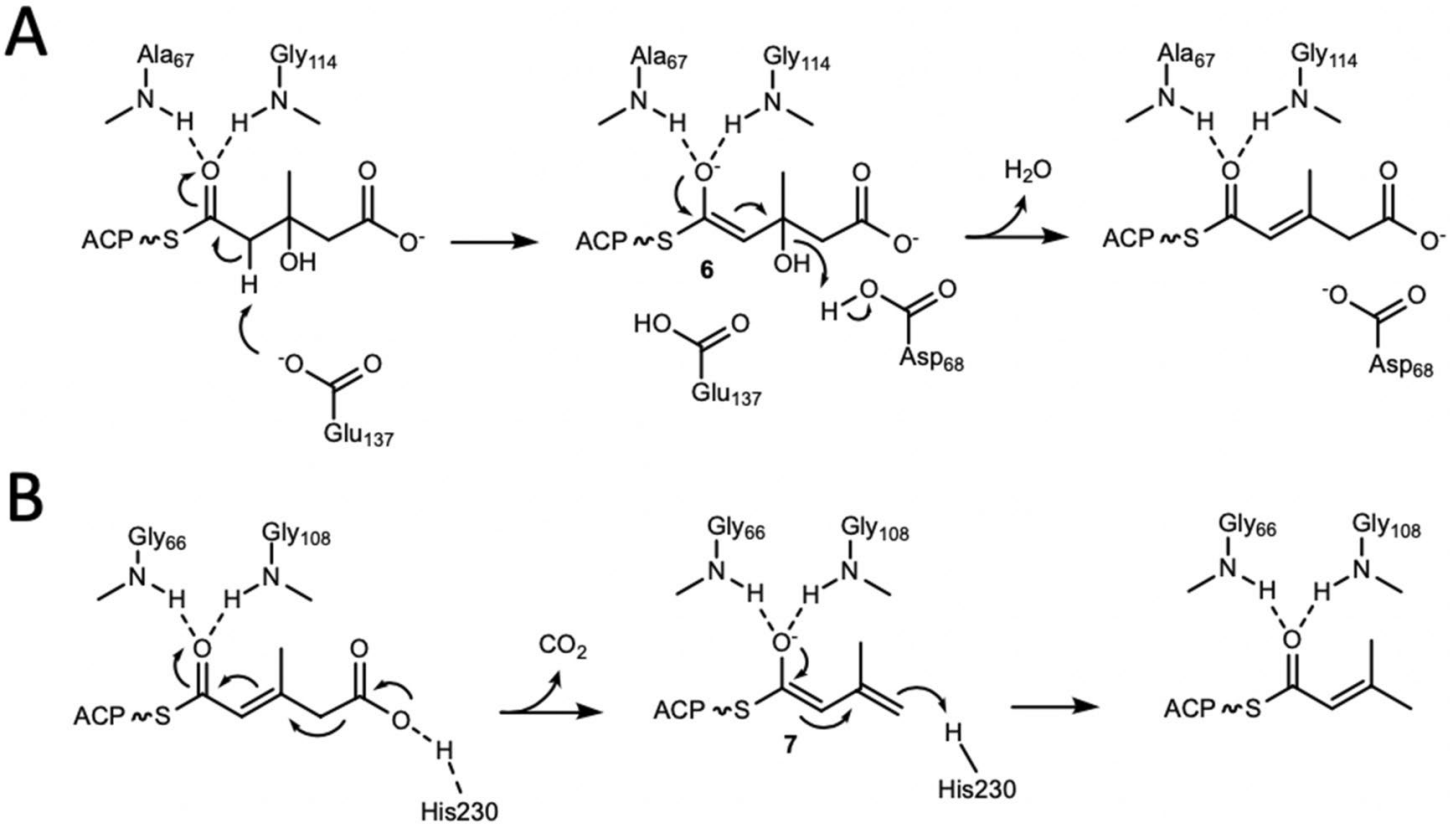
Catalytic mechanisms of PksH and PksI. (**A**) Proposed catalytic mechanism of PksH. (**B**) Proposed catalytic mechanism of PksI.

In an effort to elucidate the catalytic mechanism of PksH, the wild type (WT) enzyme, along with the point mutants PksH-D68A and PksH-E137A were produced recombinantly by overexpression in *E. coli* and purified to homogeneity using a two-step procedure (Table 1 and Materials and Methods). All proteins used for enzyme assays were of > 95% purity as judged by SDS-PAGE analysis. In the first instance, assays were attempted by monitoring the conversion of commercially sourced HMG-CoA or synthesized racemic HMG-*N*-acetylcysteamine (HMG-SNAC; see Materials and Methods) to 3-methyl glutaconyl-CoA (3MG-CoA) or its SNAC equivalent by WT PksH, using Liquid Chromatography coupled Mass Spectrometry (LCMS; Fig. 3). Both CoA and SNAC thioesters are frequently employed as surrogates of authentic ACP-bound intermediates. As racemic HMG-SNAC was used in all bioassays, it would be reasonable to expect that only one enantiomer would be recognised for dehydration. There was clear evidence of the PksH catalyzed conversion of CoA and SNAC tethered substrates to their respective glutaconyl products, as indicated by the observation of new peaks in the LCMS (Fig. 3B,C) with masses consistent with the expected products **4** (SI). However, reaction products were only observed following the incubation of assay mixes for > 72 h at 37 °C, incorporating > 5 mg/ml PksH. These data were interpreted to imply that the equilibrium of the PksH catalyzed transformation favors the reverse reaction. In an effort to circumvent this problem, we cloned and recombinantly overexpressed WT PksI in *E. coli* and purified the resulting protein to homogeneity. Reactions were repeated including recombinant WT PksI in addition to PksH, with both proteins at 1 mg/ml, and assays incubated for 16 h at 37 °C (Fig. 3). LCMS analysis of these coupled assays identified new peaks on the chromatograms, which eluted at either ~ 3.4 min (CoA) or ~ 5.5 min (SNAC) and possessed masses and retention times consistent with those of 3-methyl crotonyl-CoA (3MC-CoA) or 3-methyl crotonyl-SNAC (3MC-SNAC; Figs. 3B,C, and SI). The identities of these compounds were confirmed by comparison with synthetic standards (retention time and mass). Together, these data demonstrate that WT PksH is catalytically competent and also illustrate the value of employing the PksH/PksI coupled assay when assessing the activity of PksH and its mutants. PksH was found to accept and act upon both CoA and SNAC tethered substrates equally well under the assay conditions used. Following the successful demonstration of WT PksH activity, assays were repeated using the two PksH point mutants PksH-D68A and PksH-E137A (Fig. 4A,B). PksH-D68A was found to be active but kinetically impaired with both SNAC and CoA conjugated HMG. There was an almost complete loss of product **5** formation in assays including HMG-SNAC (Fig. 4A), and a modest reduction in product yield in assays incorporating HMG-CoA (Fig. 4B). PksH-E137A was found to be unable to catalyze the conversion of HMG-SNAC to 3-methyl glutaconyl-SNAC (Fig. 4A), and displayed significantly impaired activity with HMG-CoA, with a barely detectable quantity of crotonyl product **5** observed (Fig. 4B). These data imply a critical role for Glu137 in PksH activity. Given that both PksH mutants exhibit secondary structure compositions and hydrodynamic behaviors equivalent to that of the WT enzyme, as established by CD spectroscopy and SEC (data not shown), we conclude that the observed kinetic effects are not a consequence of protein misfolding. Based on these findings, we propose that the PksH catalyzed dehydration reaction occurs via hydrogen bonding of the thioester carbonyl by the backbone amides of Ala67 and Gly114 in the oxyanion hole and deprotonation by Glu137 to give **6** (Fig. 5A). Dehydration then occurs to give a 3-methyl glutaconyl product and water, with Asp68 functioning as a proton donor. The observed impaired activity of PksH-D68A may reflect the ability of an active site water to act as a surrogate proton source in this mutant.

**Table 1:** Primers used during this study.

Primer name	Sequence 5′–3′
PksH_WT_fwd	catatggatctcgtgacctatcaa
PksH_WT_rev	ctcgagttactgatcctccca
PksH_D68A_fwd	ggtgttttgtttcggagcgg**c**ttttcaagaaatctatcagg
PksH_D68A_rev	cctgatagatttcttgaaaa**g**ccgctccgaaacaaaacacc
PksH_E137A_fwd	tcattcagtctctctg**c**gctgctattcggcctg
PksH_E137A_rev	caggccgaatagcagc**g**cagagagactgaatga
PksI_WT_fwd	gttctgtttcagggccgatgacaaagtgacaaagtgcaatctgcctgagggt
PksI_WT_rev	atggtctagaaagctttattatgattcgcctccacatttctcaatgtt
PksI_K80A_fwd	cttcgaattcaacagggattgacc**gc**gttcactgatgacaatctttattc
PksI_K80A_rev	gaataaagattgtcatcagtgaac**gc**ggtcaatccctgttgaattcgaag
PksI_H230A_fwd	gttctgtttcagggccgatgacaaagtgacaaagtgcaatctgcctgagggt
PksI_H230A_rev	atggtctagaaagctttaattaccatataaacctttgattctacttttgacttcttcatgatgaaatgttttctca**gc**catcatcaattcc
PksI_K232A_fwd	tgaacaggaattgatgatgcatgag**gc**aacatttcatcatgaagaagtcaaa
PksI_K232A_rev	tttgacttcttcatgatgaaatgtt**gc**ctcatgcatcatcaattcctgttca
PksI_H235A_fwd	gttctgtttcagggccgatgacaaagtgacaaagtgcaatctgcctgagggt
PksI_H235A_rev	atggtctagaaagctttaattaccatataaacctttgattctacttttgacttcttcatga**gc**aaatgttttctcatgc

Bases in bold show sites of mutation.

### Crystal structure of PksI

To complement our studies of PksH we next turned our attention to its partner decarboxylase PksI. WT PksI was recombinantly overexpressed in *E. coli* and purified to homogeneity (Table 1). This polypeptide was crystallized and its structure determined using X-ray diffraction methods (Table 2). Two crystal structures of PksI were elucidated; the first to 1.93 Å resolution using a crystal cryoprotected in glycerol (PksI_GOL), and the second to 2.1 Å using a crystal cryoprotected using ethylene glycol (PksI_EG). Both structures describe an asymmetric unit comprised of three protein chains arranged in the form of a trimer (Fig. 2A). This oligomeric state is consistent with that previously reported for CurF^29^, the equivalent enzyme from the curacin *cis*-AT PKS pathway, with a Cα r.m.s.d between the two structures of 1.2 Å. SEC analysis of recombinant PksI demonstrates that this protein is trimeric both in solution and in the crystal (Fig. 2B). Each trimer exhibits a discoidal shape analogous to that of PksH, with dimensions of 83 Å × 75 Å × 39 Å. The final model of PksI_GOL comprises residues 1–247 of chain A, residues 4–249 of chain B and residues 3–231 of chain C, 663 water molecules, 6 glycerol molecules and 1 HEPES molecule. The final model of PksI_EG comprises residues 1–248 of chain A, residues 2–248 of chain B, and residues 4–232 of chain C, 417 water molecules, 4 molecules of ethylene glycol and 2 molecules of glycerol. There is a single active site per PksI monomer (Fig. 2A), with each active site positioned on the periphery of the trimer, ~ 43 Å from one other. Akin to PksH, PksI adopts a characteristic CS fold, with a ββα core, that comprises two approximately perpendicular -sheets surrounded by -helices (Fig. 2D).

**Table 2:** Summary of X-ray data collection and refinement statistics.

Data collection	PksI-Gol	PksI-EG	PksI-K80A	PksI-H230A	PksI-K232A
**Beamline**					
Wavelength (Å)	0.9762	0.9763	0.9795	0.92	0.9763
Space Group	*P*2_1_2_1_2_1_	*P*2_1_2_1_2_1_	*P*2_1_2_1_2_1_	*P*2_1_2_1_2_1_	*P*2_1_2_1_2_1_
**Cell dimensions**					
*a, b, c* (Å)	82.89, 84.66, 125.92	82.63, 84.28, 125.8	81.85, 85.69, 126.71	80.52, 85.62, 125.79	83.15, 85.26, 125.8
a = b = g (°)	90	90	90	90	90
Resolution (Å)^a^	43.1–1.93 (1.98–1.93)	52.42–2.10 (2.16–2.10)	54.6–2.1 (2.2–2.1)	50.69–2.17 (2.28–2.17)	59.53–1.75 (1.78–1.75)
*R*_merge_ (%)	13.7 (78.8)	14.2 (72.9)	7.6 (43.6)	9.0 (40.0)	6.5 (73.2)
Total no. of reflections	943,624 (63,758)	743,179 (61,734)	183,013 (25,388)	232,589 (34,358)	538,491 (27,268)
No. of Unique reflections	67,335 (4,466)	52,072 (4,234)	52,523 (7,446)	46,135 (6,699)	90,710 (4,426)
Average *I*/*I*	16.4 (3.7)	15.3 (4.3)	11.3 (2.6)	11.5 (3.4)	13.4 (2.4)
CC_1/2_ (%)^a^	100 (92.6)	99.9 (89.9)	100 (83.7)	99.9 (85.4)	99.9 (89.7)
Completeness (%)	100 (100)	100 (100)	98.1 (96.6)	98.4 (99.4)	99.9 (99.8)
Redundancy	14.0 (14.3)	14.3 (14.6)	3.5 (3.4)	5.0 (5.1)	5.9 (6.2)
**Refinement**					
*R*_work_/*R*_free_ (%)	15.1/18.5	16.7/21.4	19.8/25.2	17.5/23.0	16.6/19.3
**No. atoms**					
Protein	5,886	5,627	5,267	5,765	5,804
Water	497	320	233	258	542
**Average B-factor** (Å^2^)					
Protein	18.97	23.01	27.60	26.27	25.36
Water	31.97	37.07	45.10	35.11	39.06
**Rms deviations**					
Bond lengths (Å)	0.025	0.021	0.02	0.019	0.023
Bond angles (°)	2.231	2.085	2.020	2.071	2.240
Ramachandran favored (%)	97.4	96.0	94.1	94.9	97.2
Ramachandran outliers (%)	0	0.4	1.7	1.0	0.2
PDB code	4Q1H	4Q1G	4Q1I	4Q1J	4Q1K

^a^Values in parentheses are for highest-resolution shell.

### Active architecture of site of PksI

The active site of PksI is located at the base of a solvent exposed channel that permits access to the catalytic machinery of the enzyme. The base of the channel houses a single histidine residue, His230, which occupies an equivalent position to His240 in CurF (Figs. 2F and S1). Based on this positioning His230 would be predicted to be the catalytic histidine of PksI. A histidine at this position is universally conserved in all other -methyl branching decarboxylases for which sequences have been reported. In addition to His230 the active site of PksI comprises the residues Phe81, Phe136, Phe234 and His235. In the PksI_GOL structure a single monomer (chain A) within the PksI trimer possess a bound glycerol molecule within its active site. Comparison of the active site architecture of PksI monomers in glycerol bound and free states allows assessment of active site reorganization as a consequence of ligand binding (Fig. 2F). In the unliganded structure, the active site adopts an “closed” conformation, with the side chains of Phe136 and Phe81 pointing directly into the centre of the active site cavity and His230 rotated to face away from the central chamber (Fig. 2F). In the liganded structure, the active site is occupied by the substrate mimic glycerol, whose hydroxyl group can form a hydrogen bond with His230. For glycerol to be accommodated within the active site of PksI, the side chains of residues in this region must be reconfigured accordingly. In this “open” conformation the side chains of Phe136 and Phe81 are seen to flip away from the centre of the active site, which is occupied by the bound glycerol molecule. As a consequence, Phe136 forms a tripartite stacking interaction with Phe234 and His235, which sits beneath the proposed catalytic residue His230, which itself rotates to point directly into the centre of the active site. It is likely that our unliganded structure represents the ground state of the enzyme, with the glycerol bound form equating to a mimic of a PksI reaction intermediate. The observed side chain rearrangements in the glycerol bound structure are essential for allowing the substrate to be accommodated within the catalytic site of the enzyme and may also function to promote active site solvation, consistent with a potential role for water as a proton donor during catalysis. In the PksI_EG structure all the active sites adopt the “closed” ground state conformation.

### Enzyme assays and catalytic mechanism of PksI

To extend our structural studies of PksI, we next sought to elucidate the catalytic mechanism of this enzyme. Kinetic assays were performed using the same coupled assay system as that developed to study PksH, employing either commercially sourced HMG-CoA or synthesized racemic HMG-SNAC as substrates (Fig. 3). Assays were conducted using WT PksI, along with the point mutants PksI-H230A, PksI-H235A, PksI-K80A, PksI-K232A. Although our structural data strongly implied that His230 was the catalytic histidine in the PksI reaction, the positioning of His235 on the periphery of the enzyme active site meant that a role for this residue in catalysis could not be discounted. The two lysine mutants were generated in an effort to identify the proton donor for the PksI catalyzed reaction. In the proposed decarboxylation mechanism of CurF, an active site lysine was shown to be critical for enzyme activity^29^. Based on our available crystal structures both Lys80 and Lys232 were identified as potential candidates for this role in PksI. WT and mutant proteins were produced recombinantly by overexpression in *E. coli* and purified to homogeneity using a two-step procedure. All proteins used for enzyme assays were of > 95% purity as judged by SDS-PAGE analysis. As reported above PksH/PksI coupled assays gave reaction products with retention properties and masses consistent with the predicted crotonyl products **5** (Fig. 3 and SI). In all cases HMG-CoA was found to be a better starting substrate for PksI (WT and mutants) than HMG-SNAC, implying a preference for longer chain substrate mimetics (Fig. 3B,C).

Of the four PksI mutants investigated only PksI-H230A was found to be catalytically compromised (Fig. 4C,D), with barely detectable activity for 3-methyl glutaconyl-SNAC and significantly impaired activity for 3-methyl glutaconyl-CoA. This observation is consistent with the proposed key role of this amino acid in the decarboxylation reaction. Given that PksI-H235A showed in vitro activity equivalent to that of the WT enzyme, a role for this residue in PksI catalysis can be discounted. Surprisingly, PksI-K80A and PksI-K232A both display catalytic activities akin to that of WT-PksI, with no evidence of functional impairment. Together, these data imply a deviant mechanism of catalysis in PksI distinct from that previously reported for CurF. This is unsurprising given the disparities between the different active site architectures of these two enzymes. In the absence of any other potential proton donors within the PksI active site, we thus propose a variant mechanism of decarboxylation in which His230 functions as both an acid and a base at physiological pH (Fig. 5B). It is unclear at this stage as to the origin of PksI-H230A activity for the CoA tethered substrate, with the observed turnover (albeit reduced) likely arising from the presence of an active site water molecule.

In tandem with kinetic assays, the crystal structures of PksI-K80A, PksI-K232A and H230A were determined (Table 2). The active sites of each of these enzymes displays the ground state “closed” conformation as observed in the WT enzyme, with no evidence of significant structural perturbations within the active site. These data thus imply that the observed kinetic effects are not attributable to global or local structural reorganization within the enzyme as a consequence of the amino acid substitutions made.

### PksH and PksI do not form heterotrimeric complexes

Given their significant structural identities we hypothesized that PksH and PksI may co-assemble to form heterotrimeric complexes. Such an organisation would provide an elegant solution to achieving spatial colocation of dehydration and decarboxylation activities during β-methyl branch processing. In a bid to establish if PksH and PksI were able to form heterotrimers, these proteins were co-expressed in *E. coli* and the resulting material purified to homogeneity. The expression system used was chosen to enable the selection of dual *pks*I/*pks*H transformants, using the antibiotics carbenicillin (*pks*I::pOPINF) and kanamycin (*pks*H::pET28A), followed by recombinant co-expression and protein purification. Although both constructs encode N-terminally hexa-his tagged proteins, these tags may be cleaved using different proteases; PksH thrombin cleavable, PksI C3 cleavable. It is therefore possible to selectively cleave the his-tag from either protein while leaving an intact tag fused to the other, thus enabling the identification and recovery of heterotrimeric complexes using nickel affinity chromatography.

**Figure 6:**
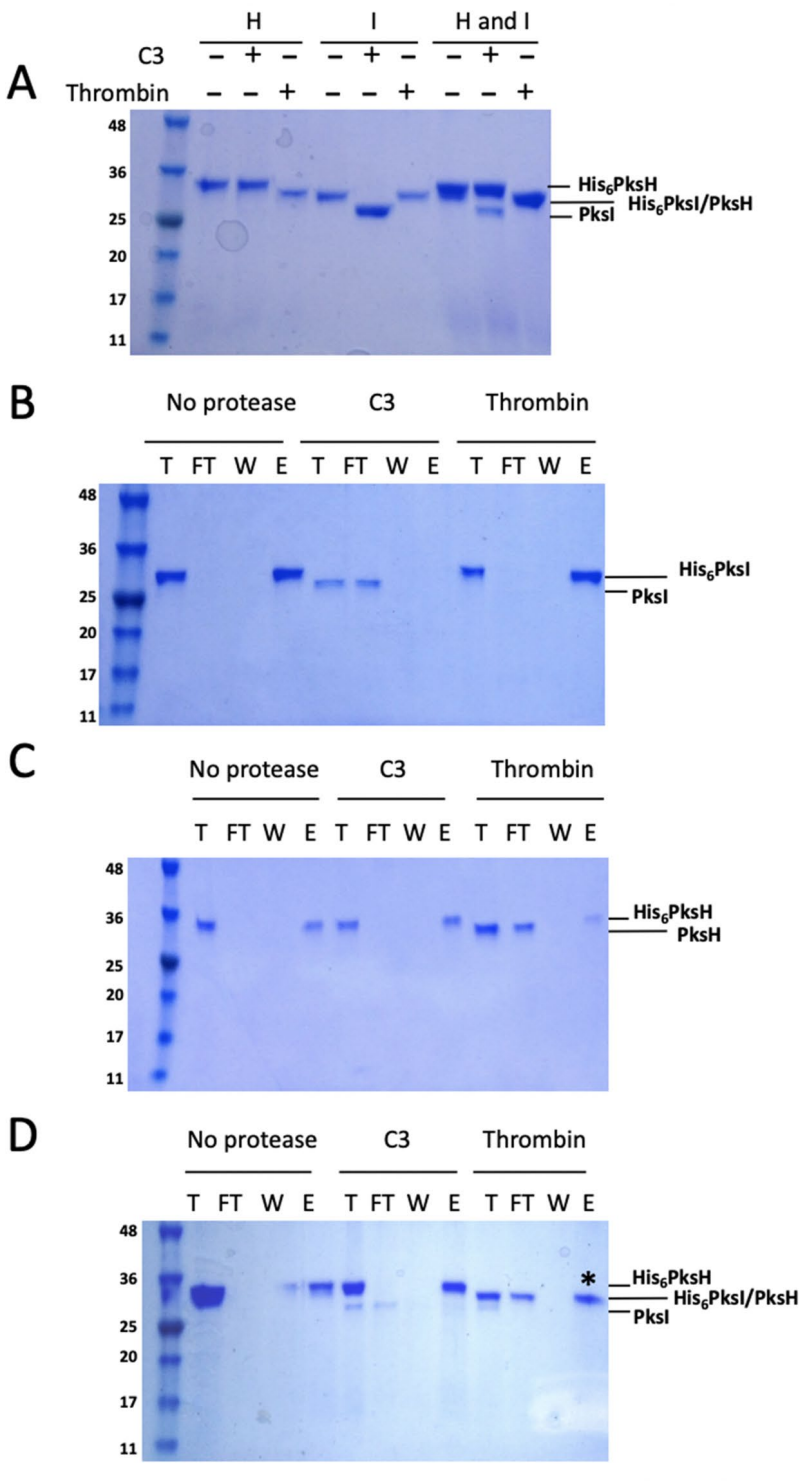
Co-expression and pull-down assays for PksH/PksI heterotrimer formation. Co-expressed and purified His_6_PksH and His_6_PksI were treated with C3 protease (to cleave His_6_PksI) or thrombin protease (to cleave His_6_PksH), then repurified by Ni^2+^-affinity. (**A**) SDS-PAGE gel showing the test digestion of PksH, PksI and coexpressed PksH/PksI with 3C protease and thrombin. + / − signs indicate the presence or absence of the respective protease. (**B**) SDS-PAGE gel showing PksI protease digests and subsequent Ni^2+^-affinity repurification. (**C**) SDS-PAGE gel showing PksH protease digests and subsequent Ni^2+^-affinity repurification. (**D**) SDS-PAGE gel showing protease digests and subsequent Ni^2+^-affinity repurification coexpressed PksH-PksI. The band marked * was analyzed by tryptic digest MALDI MS and was identified as PksI only. Labels correspond to the following; total protein loaded (T), column flow-through (FT), column wash (W) and column eluate (**E**). All gel images were collected using a BioDoc-It imaging system (UVP Ltd.) with integrated Doc-It LS Analysis software version 1 (https://www.uvp.com/) applying the system’s default imaging settings.

In the first instance control experiments were conducted to demonstrate that selective his-tag cleavage could be achieved. Treatment of recombinant his-tagged PksH with thrombin results in a small mass change observable by SDS-PAGE consistent with tag removal, with analogous behavior observed upon treatment of PksI with C3 protease (Fig. 6A). Treatment of PksH with C3 protease and PksI with thrombin, results in no mass change consistent with no tag cleavage (Fig. 6A). These data illustrate target specific activity for the proteases employed in his study. Next, protease treated samples were subjected to HisPur Ni–NTA Spin Column repurification in an effort to establish their capacity to bind immobilized nickel ions. Both untreated and thrombin treated PksI bound to the Ni–NTA column matrix and showed no reduction in molecular weight (Fig. 6B). In contrast, C3 treated PksI was not retained on the column and exhibited a reduction in mass consistent with his-tag removal. Equivalent results were obtained when this experiment was repeated substituting PksI for PksH (Fig. 6C). In this instance, his-tag cleavage and his-load buffer elution is only observed for protein samples pretreated with thrombin. Co-expressed and purified PksH/PksI was subjected to the same protease digestion regime as outlined above (Fig. 6D). In the absence of protease both polypeptides are observed in the column eluent only. When incubated with C3 protease a clear reduction in mass is observed consistent with tag cleavage from PksI, but not from PksH (Fig. 6A). When this material is subjected to HisPur Ni–NTA repurification a clear band is observed in the column flow through, consistent with non-retention of PksI (Fig. 6D). Following treatment with elution buffer, however, a single band is observed by SDS-PAGE in the eluate with a molecular weight consistent with that of PksH (Fig. 6D). There is no evidence of PksI in this sample, and by extension, no evidence of PksH/PksI heterotrimer formation. Treatment of co-expressed and purified PksH/PksI with thrombin results in a clear reduction in the mass of PksH (Fig. 6A). As his-tag cleaved PksH possesses the same molecular weight as uncleaved PksI a single band is observed on the gel. Repurification of this material gave a single species with a molecular weight consistent with that of tag cleaved PksH, implying that this protein is not retained on the column following thrombin treatment. SDS-PAGE analysis of the eluted sample reveals a single species with a molecular weight consistent with non-tag cleaved PksI. Given that it is not possible to distinguish between tag cleaved PksH and uncleaved PksI this band was subjected to tryptic digest MALDI MS and was identified as PksI only (Figures S2 and S3). Together, these data demonstrate that PksH and PksI do not form heterotrimeric complexes, even when co-expressed and purified.

### Discussion

In this study we present a structural and functional description of the bacillaene synthase β-branching enzymes PksH and PksI. These biocatalysts are responsible for the final two steps in β-methyl branch incorporation in this PKS/NRPS pathway. Crystals structures of both enzymes, in combination with in vitro enzyme assays using synthesized substrate analogues and mutagenesis studies, have provided insight into the catalytic mechanisms of these two enzymes. Our findings constitute the first reported molecular description of a HCS cassette dehydratase, and identify a deviant catalytic mechanism in PksI, which is distinct from that previously reported in other HCS cassette decarboxylases.

Our structural studies demonstrate that both PksH and PksI adopt distinctive trimeric CS folds, both in vitro and *in crystallo*. The active sites of both enzymes (one per monomer) are situated in equivalent locations on the periphery of their respective oligomers. The chemically disparate nature of the dehydration and decarboxylation reactions catalyzed by these polypeptides serves to further highlight the diverse array of chemistries that can be supported by CS members. Intriguingly, despite possessing analogous folds and trimeric architectures, we have been unable to provide any evidence that these polypeptides can co-assemble to form heterotrimeric complexes, even when heterologously co-expressed in *E. coli*. This is likely to be a direct consequence of the incompatibility of the amino acid residues that occupy the trimer interfaces of either protein. Significantly, in these regions the two polypeptides share < 10% primary amino acid sequence identity. This reflects the disparate residue composition of both proteins at their respective trimer interfaces and offers an explanation for their inability to co-assemble into a single functional unit.

PksH has been shown herein to catalyze the dehydration of HMG substrates in vitro. However, the low product yield appears to reflect the unfavourable equilibrium of this reaction. Through the development of a PksH/PksI coupled assay, it has proven possible to investigate the catalytic activity of this enzyme and guided by the available crystal structure, in tandem with mutagenesis studies, we have probed the dehydration mechanism of this protein. These data show that catalysis by PksH is dependent on the residue Glu137 and is impacted by substitution of Asp68 with alanine, implying a potential role for this latter amino acid as a proton donor in the PksH catalyzed reaction. In keeping with other CS family members, this mechanism is enabled through the polarization of the substrate thioester carbonyl by an active site oxyanion hole, resulting in formation of intermediate **6** (Fig. 5A).

In addition to our studies of PksH, we have also investigated the structure and mechanism of its partner decarboxylase PksI. The activity of this enzyme has been demonstrated in vitro using the same coupled assay system as that employed for PksH. Structure guided mutagenesis and enzyme assays have been used to delineate the catalytic mechanism of this polypeptide. The PksI reaction is shown herein to be dependent on the single catalytic residue His230, indicating a mechanism distinct from that proposed in the curacin *cis*-AT branching decarboxylase CurF. In CurF, an active site lysine was identified as the proton donor^29^. However, in PksI, mutation of the two candidate lysine residues in the active site of this enzyme to alanines yielded proteins with activities equivalent to that of the WT protein. Crystal structures of the mutants PksI_K80A, PksI K232A and PksI H230A revealed no significant changes in active site architecture beyond side chain identity. We thus propose a His230 dependent mechanism wherein this residue has a dual function both catalyzing decarboxylation and to act as a proton donor to the decarboxylated intermediate **7** to give the reaction product (Fig. 5B). Progression of the reaction is facilitated by stabilization of the intermediate by an oxyanion hole formed by the backbone amides of Gly66 and Gly108.

Structural studies of WT PksI have also serendipitously revealed that substrate binding is accompanied by significant reorganisation of the enzyme active site. In the absence of bound ligand, the PksI active site adopts an “closed” conformation, where the side chains of residues Phe81 and Phe136 occupy the central solvent exposed cavity of the enzyme active site. In this state the catalytic residue His230 is found to point away from the centre of the site in an apparent catalytically incompetent conformation. Upon ligand binding, the side chains of Phe81 and Phe136 rotate away to enable substrate to be accommodated in the active site pocket. In this conformation Phe234 forms a π–π stacking interaction with Phe136, and the side chain of His230 rotates to present the imidazole N1 for hydrogen bonding to the substrate terminal hydroxyl. The high proportion of aromatic amino acid content within the PksI active site is not shared in other HCS cassette decarboxylases, which implies that the observed ligand induced active site reorganisation represents a distinct binding mode in this enzyme. Unlike PksH, which displays no preference for CoA over SNAC tethered substrates, or vice versa, PksI shows significantly greater in vitro activity when using HMG-CoA as opposed to HMG-SNAC as the coupled assay starting substrate. This indicates a requirement for longer chain substrates in this enzyme. This is unsurprising, given that the authentic substrate for both the dehydration and decarboxylation reactions would be presented on the phosphopantetheine arm of either of the PksL di-domain ACPs. It is also important to emphasise that in this context protein–protein interactions between both PksH and PksI, and the substrate presenting ACPs, will significantly influence the rate and processivity of methyl branch incorporation. This may act to negate any requirement for the coordinated recruitment of PksH and PksI to the branching ACPs as indicated by their inability to co-assemble into a single functional oligomer.

In summary, the data presented herein reveal the molecular intricacies of the final steps in β-methyl branch incorporation in bacillaene biosynthesis, and further expand the mechanistic diversity of enzyme chemistries that can be supported by CS family polypeptides. It is anticipated that these findings will prove valuable in informing future efforts in the exploitation of PksH/PksI and related biocatalysts for the targeted introduction of methyl branches into unbranched polyketides, further expanding the natural product repertoire and facilitating access to new bioactive scaffolds.

## Methods

### Gene cloning and site-directed mutagenesis

PksH was amplified from *B. subtillis* 168 genomic DNA by PCR and cloned into pET28a using NdeI and XhoI restriction sites yielding the construct *pks*H::pET28A. PksI was amplified *B. subtillis* 168 genomic DNA and cloned into pOPINF^31^ by ligation independent cloning (LIC) using the In-Fusion HD Cloning System (Clontech) yielding the construct *pks*I::pOPINF. Point mutations were made either using the QuikChange kit (Agilent Technologies; PksH-D68A, PksH-E137A and PksI-K232A) or by overlap extension PCR followed by re-cloning back into pOPINF using the same approach as for the WT enzyme (PksI-K80A). PksI H230A and H235A were made by a single round of PCR with a long reverse primer covering the mutation and LIC homology site; the resulting PCR product was then cloned back into pOPINF as above. The sequences of all constructs were verified by DNA sequencing prior to protein expression studies.

### Protein expression and purification

Constructs were transformed into *E. coli* BL21(DE3) cells and grown at 37 °C in 1 L of LB medium supplemented with 50 µg/ml kanamycin (PksH) or 100 µg/ml ampicillin (PksI) to an OD_600nm_ of 0.6–0.8 in 2.5 L flasks. The cultures were adjusted to 18 °C, and isopropyl β-d-thiogalactopyranoside (IPTG) added to final concentration of 1 mM and allowed to grow for an additional 16 h with shaking. Cells were harvested by centrifugation, and cell pellets frozen immediately and stored at − 80 °C. Cell pellets were defrosted and resuspended in 20 mM Tris–HCl, 500 mM NaCl, pH 7.5, 10% glycerol (PksH) or 50 mM Tris–HCl, 150 mM NaCl, pH 7.5 (PksI). Cells were lysed by sonication and clarified by centrifugation. Cell extracts were loaded onto a 5 ml HisTrap column (GE Healthcare) and washed with resuspension buffer supplemented with 20 mM imidazole. Wild type and mutant PksH proteins were eluted using a linear gradient of 0–50% his-load to his-elute buffer (20 mM Tris–HCl pH 7.5, 500 mM NaCl, 250 mM imidazole, 10% glycerol) over ten column volumes. Eluted fractions found to contain protein of the correct molecular weight, as judged by SDS-PAGE analysis, were pooled, concentrated, and loaded onto a Superdex 200 16/60 column (GE Healthcare) equilibrated with 20 mM Tris–HCl, 500 mM NaCl, pH 7.5, 10% glycerol. Wild type and mutant PksI proteins were purified using the same method as for PksH proteins, with the exception that the SEC elution buffer used was 20 mM Tris–HCl, 150 mM NaCl, pH 7.5. For both PksH and PksI proteins polypeptide containing fractions eluted from the SEC column were identified by SDS-PAGE analysis, following which fractions were pooled and concentrated to 10 mg/ml before flash freezing in liquid nitrogen and storage at − 80 °C.

### Protein crystallization, X-ray diffraction data collection and structure determination

Crystals of WT-PksI, PksI-K80A, PksI-K232A and PksI-H230A were grown using the hanging drop vapor diffusion method. Reservoir conditions used were as follows; WT-PksI, 13–18% PEG 40,000, 0.1 M MES/0.1 M imidazole, 20% glycerol, pH 6.5–8.0, or 6–16% PEG 8,000, 0.1 M MOPS/0.1 M HEPES, 20% ethylene glycol, pH 6.5–8.0; PksI-K80A, 15–25% PEG 8 K, 20% ethylene glycol, pH 6.5–8.0; PksI-K232A, 15–18% PEG 4,000, 0.1 M HEPES, 20% glycerol, pH 6.5–7.5; PksI-H230A, 12–14% PEG 8 K, 1.0 M HEPES/MOPS, 20% ethylene glycol, pH 7.5. Where necessary crystals were cryopreserved in reservoir solution supplement with either 20% glycerol or 20% ethylene glycol prior to flash freezing in liquid nitrogen. Diffraction data were collected at Diamond Light source on beam line I04, with crystals maintained at cryogenic temperatures throughout. Data were processed with MOSFLM^32^ and scaled with SCALA^33^ as implemented in the CCP4 suite of programs^34^. 5% of the data were set aside for the calculation of *R*_free_. The initial structure of PksI was determined by molecular replacement using MOLREP^35^. The search model comprised a trimer of the crystal structure of CurF (PDB entry 2Q2X;^29^. The initial model was built using Arp/WARP^36^, followed by iterative cycles of manual rebuilding and refinement using COOT^37^ and REFMAC5^38^ to produce the final model, PksI_GOL. Structures of PksI-WT cryopreserved in ethylene glycol (PksI_EG), PksI-K80A, PksI-K232A and PksI-H230A were determined by molecular replacement using the PksI_GOL structure as the search model, employing the same strategy as that used for PksI_GOL. For analysis, MOLPROBITY^39^ and LSQMAN^40^ were used to generate Ramachandran plots and superimposed structures from which root-mean-square deviations (r.m.s.d.) based on Cα atoms were determined. Protein structure figures have been prepared with PYMOL (DeLano Scientific, USA).

### Analytical size exclusion chromatography

100 µl aliquots of PksH or PksI at a concentration of 0.5 mg/ml were injected onto a Superdex 200 10/60 column pre-equilibrated with 50 mM Tris–HCl, 150 mM NaCl, 5% glycerol, pH 7.5. Elution was performed at 1 ml/min over 3 column volumes. Elution profiles were compared to those of known standards as detailed in Fig. 2B.

### Enzyme activity assays

1 mg/ml PksH and/or PksI, or mutants thereof, was incubated at 37 °C overnight with 1 mg/ml of 3-hydroxyl-3-methylglutaryl-CoA (HMG-CoA) or an *N*-acetylcysteamine derivative of HMG (HMG-SNAC) in a buffer containing 20 mM Tris–HCl, 150 mM NaCl, 10% glycerol, pH 7.5. The protein was precipitated with 2 volumes of acetonitrile and removed by centrifugation (20 min 13,000 rpm). The acetonitrile was removed by Speed-Vac and remaining substrate/product redissolved in the original volume of water. Assay samples were analyzed by LCMS using a Waters 2,767 HPLC linked to a Waters ZQ mass spectrometer. SNAC derivatives were separated on a Kinetex 2.6 µm, C18, 100 Å (Phenomenex) at 1 ml/min, using a 10 min gradient from 10 to 90% acetonitrile with the addition of 0.05% formic acid and detected using a Waters 996 diode array detector between 210 and 400 nm and the mass spectrometer scanning an m/z range between 150 and 600 Da. CoA derivatives were analysed using a similar method, except that the aqueous buffer was water with 25 mM ammonium acetate and 0.5% acetic acid, and the m/z range set to 750–950 Da.

### Co-expression and co-purification of PksH and PksI

*pks*H::pET28A and *pks*I::pOPINF were co-transformed into *E. coli* BL21(DE3) cells by heat shock, with dual transformants selected on LB agar plates supplemented with 50 µg/ml kanamycin and 100 µg/ml ampicillin. Single colonies were picked and grown at 37 °C with shaking in 1 L of LB medium supplemented with antibiotics to an OD_600nm_ = 0.6–0.8. Protein expression was induced by the addition of IPTG to a final concentration of 1 mM, and cells grown for a further 3 h at 37 °C with shaking. Cells were harvested by centrifugation and processed as outlined for PksH and PksI proteins. Recombinant protein produced from dual *pks*H/*pks*I transformants was purified as detailed for wild type PksI.

### Affinity tag cleavage assays

100 µl aliquots of wild type PksH, PksI and PksH/PksI, all at 1 mg/ml, were incubated with either thrombin or 3C protease (both GE Healthcare) to a final concentration of 10 U protease/mg protein. PksH/PksI samples were prepared either by mixing the independently purified proteins or by co-expression and co-purification. Samples were incubated for 18 h at 4 °C. Following incubation, the samples were passed through HisPur Ni–NTA spin columns (0.2 ml; Thermo Scientific). The column was washed twice with 100 µl of his-wash buffer (20 mM Tris–HCl, 150 mM NaCl, pH 7.5,) followed by an elution step using his-elute buffer (20 mM Tris–HCl, 150 mM NaCl, 500 mM imidazole, pH 7.5). Column eluents from all load, wash and elute steps were collected and visualized by SDS-PAGE.

### Trypsin digest and MALDI-TOF analysis

Samples were digested using the In-gel Tryptic Digestion Kit (Thermo Scientific) according to the manufacturer’s instructions. The tryptic digest mixture was dried and resuspended in 50:50 acetonitrile:water, then mixed with an equal volume of α-cyano-4-hydroxycinnamic acid ionisation matrix (α-CHCA) and applied to a Bruker Daltonics UltrafleXtreme 2 mass spectrometer operated in positive ion reflection mode. The results were analysed using MASCOT Peptide Mass Fingerprint (Matrix Science), searching the NCBInr database with fixed carbamidomethyl modification and variable oxidation modification, and a peptide tolerance of ± 200 ppm.

### Synthesis of PksH SNAC substrate 3 and product 5

#### (2′-Acetamidoethyl)thio) 3-hydroxy-3-methylpentanthioate 3


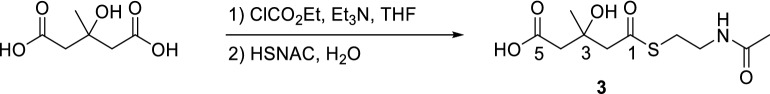


3-Hydroxy-3-methyl-1,3-dioic acid (0.323 g, 2.56 mmol) was dissolved in dry THF (20 mL) at 0 °C, under nitrogen. Et_3_N (0.36 mL, 2.56 mmol) and chloroformate (0.25 mL, 2.56 mmol) were added. The mixture was stirred for 1 h before the precipitated Et_3_NHCl was removed by filtration. The filtrate was added dropwise over 1 h to a solution of HSNAC (0.244 g, 2.04 mmol) in water (3 mL) at 0 °C under nitrogen. The pH was maintained at 7.0 by addition of 0.1 M NaOH. The reaction was stirred for 2 h before 3 M HCl solution was added to lower the pH to 1. The solution was extracted with ethyl acetate (3 × 20 mL), and the aqueous layer was lyophilized. The residue was then dissolved in methanol (2 mL), filtered and the solvent removed *in vacuo* to yield **3** (135 mg, 25%); δ_H_ (400 MHz, D_2_O) 1.37 (3H, s, CH_3_), 1.94 (3H, s, NCOCH_3_), 2.66 (2H, s, 4-H_2_), 2.98 (2H, s, 2-H_2_), 3.04 (2H, t, *J* 6.5, NCH_2_), 3.35 (2H, t, *J* 6.5, SCH_2_); δ_C_ (100 MHz, D_2_O) 21.9 (NCO*C*H_3_), 26.4 (CH_3_), 28.3 (NCH_2_), 38.5 (SCH_2_), 45.1 (C-4), 53.6 (C-2), 70.3 (C-3), 174.3 (C=O), 174.8 (C=O), 200.1 (C-1).

### *S*-(2′-Acetamidoethyl) 3-methylbut-2-enethioate 5


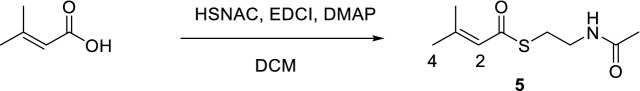


To a stirred solution of 3,3-dimethylacrylic acid (0.550 g, 5.5 mmol) in dry DCM (20 mL) at 0 °C under nitrogen was added EDCI.HCl (1.089 g, 5.68 mmol) and DMAP (70 mg, 0.55 mmol). After stirring at 0 °C for 15 min, HSNAC (0.677 g, 5.68 mmol) was added in dry DCM (5 mL). The mixture was allowed to warm to room temperature and stirred overnight. The reaction mixture was then washed successively with 0.01 M HCl (3 × 30 mL) and brine (30 mL), then dried over MgSO_4_, filtered and the solvent removed *in vacuo*. The residue was purified by flash chromatography (5% MeOH/DCM) to give **5** (1.288 g, 93%) as a colourless oil which slowly solidified. δ_H_ (400 MHz, CDCl_3_) 1.89 (3H, d, *J* 1.5, 4-H_3_), 1.95 (3H, s, NCOC*H*_3_), 2.15 (3H, d, *J* 1.5, CH_3_), 3.04 (2H, t, *J* 6.5, SCH_2_), 3.44 (2H, q, *J* 6.5, NCH_2_), 5.95 (1H, br s, NH), 6.00 (1H, dt, *J* 2.6, 1.5); δ_C_ (100 MHz, CDCl_3_) 21.2 (CH_3_), 23.0 (NCO*C*H_3_), 27.2 (C-4), 28.3 (SCH_2_), 39.8 (NCH_2_), 122.9 (C-2), 154.6 (C-3), 170.5 (N*C*OCH_3_), 189.2 (C-1); Found (CI): 202.0896 [MH]^+^, (C_9_H_16_O_2_NS requires 202.0902). Spectroscopic data in accordance with the literature^21^.

## Supplementary information

Supplementary Information 1.

## Data Availability

Atomic coordinates and structure factors for PksI-EG, PksI-GOL, PksI_K80A, PksI_H230A and PksI_K232A have been deposited with the PDB with accession codes 4Q1G, 4Q1H, 4Q1I, 4Q1J, and 4Q1K respectively.

## References

[1] Staunton J, Weissman KJ (2001). Polyketide biosynthesis: a millennium review. Nat. Prod. Rep..

[2] Weissman KJ (2009). Chapter 1 introduction to polyketide biosynthesis. Methods Enzymol..

[3] Marinelli F (2009). Chapter 2 from microbial products to novel drugs that target a multitude of disease indications. Methods Enzymol..

[4] Fischbach MA, Walsh CT (2006). Assembly-line enzymology for polyketide and nonribosomal peptide antibiotics: logic machinery, and mechanisms. Chem. Rev..

[5] Till M, Race PR (2016). The assembly line enzymology of polyketide biosynthesis. Methods Mol. Biol..

[6] Keating TA, Walsh CT (1999). Initiation, elongation, and termination strategies in polyketide and polypeptide antibiotic biosynthesis. Curr. Opin. Chem. Biol..

[7] Hertweck C (2009). The biosynthetic logic of polyketide diversity. Angew. Chem. Int. Ed. Engl..

[8] Walsh CT (2004). Polyketide and nonribosomal peptide antibiotics: modularity and versatility. Science.

[9] Walsh CT (2008). The chemical versatility of natural-product assembly lines. Acc. Chem. Res..

[10] Sundaram S, Hertweck C (2016). On-line enzymatic tailoring of polyketides and peptides in thiotemplate systems. Curr. Opin. Chem. Biol..

[11] Helfrich EJ, Piel J (2016). Biosynthesis of polyketides by trans-AT polyketide synthases. Nat. Prod. Rep..

[12] Piel J (2010). Biosynthesis of polyketides by trans-AT polyketide synthases. Nat. Prod. Rep..

[13] Calderone CT, Kowtoniuk WE, Kelleher NL, Walsh CT, Dorrestein PC (2006). Convergence of isoprene and polyketide biosynthetic machinery: isoprenyl-S-carrier proteins in the pksX pathway of *Bacillus subtilis*. Proc. Natl. Acad. Sci. USA.

[14] Walker PD (2019). Control of β-Branching in kalimantacin biosynthesis: application of ^13^C NMR to polyketide programming. Angew. Chem. Int. Ed. Engl..

[15] Calderone CT (2008). Isoprenoid-like alkylations in polyketide biosynthesis. Nat. Prod. Rep..

[16] Miziorko HM (2011). Enzymes of the mevalonate pathway of isoprenoid biosynthesis. Arch. Biochem. Biophys..

[17] Haines AS (2013). A conserved motif flags acyl carrier proteins for β-branching in polyketide synthesis. Nat. Chem. Biol..

[18] Till M, Race PR (2014). Progress challenges and opportunities for the re-engineering of *trans*-AT polyketide synthases. Biotechnol. Lett..

[19] Butcher RA (2007). The identification of bacillaene, the product of the PksX megacomplex in *Bacillus subtilis*. Proc. Natl. Acad. Sci. USA.

[20] Moldenhauer J, Chen XH, Borriss R, Piel J (2007). Biosynthesis of the antibiotic bacillaene, the product of a giant polyketide synthase complex of the trans-AT family. Angew. Chem. Int. Ed. Engl..

[21] Jensen K (2012). Polyketide proofreading by an acyltransferase-like enzyme. Chem. Biol..

[22] Nguyen T (2008). Exploiting the mosaic structure of trans-acyltransferase polyketide synthases for natural product discovery and pathway dissection. Nat. Biotechnol..

[23] Chen XH (2006). Structural and functional characterization of three polyketide synthase gene clusters in *Bacillus amyloliquefaciens* FZB 42. J. Bacteriol..

[24] Straight PD, Fischbach MA, Walsh CT, Rudner DZ, Kolter R (2007). A singular enzymatic megacomplex from *Bacillus subtilis*. Proc. Natl. Acad. Sci. USA.

[25] Patel PS (1995). Bacillaene, a novel inhibitor of procaryotic protein synthesis produced by *Bacillus subtilis*: production, taxonomy, isolation, physico-chemical characterization and biological activity. J. Antibiot..

[26] Maloney FP, Gerwick L, Gerwick WH, Sherman DH, Smith JL (2016). Anatomy of the β-branching enzyme of polyketide biosynthesis and its interaction with an acyl-ACP substrate. Proc Natl Acad Sci USA.

[27] Buchholz TJ (2010). Polyketide β-branching in bryostatin biosynthesis: identification of surrogate acetyl-ACP donors for BryR, an HMG-ACP synthase. Chem. Biol..

[28] Gu L (2009). Metamorphic enzyme assembly in polyketide diversification. Nature.

[29] Geders TW (2007). Crystal structure of the ECH2 catalytic domain of CurF from *Lyngbya majuscula*. Insights into a decarboxylase involved in polyketide chain beta-branching. J. Biol. Chem..

[30] Hamed RB, Batchelar ET, Clifton IJ, Schofield CJ (2008). Mechanisms and structures of crotonase superfamily enzymes–how nature controls enolate and oxyanion reactivity. Cell. Mol. Life Sci..

[31] Berrow NS (2007). A versatile ligation-independent cloning method suitable for high-throughput expression screening applications. Nucleic Acids Res..

[32] Leslie A (2006). The integration of macromolecular diffraction data. Acta Crystallogr. D Biol. Crystallogr..

[33] Evans P (2006). Scaling and assessment of data quality. Acta Crystallogr. D Biol. Crystallogr..

[34] Winn MD (2011). Overview of the CCP4 suite and current developments. Acta Crystallogr. D Biol. Crystallogr..

[35] Vagin A, Teplyakov A (2010). Molecular replacement with MOLREP. Acta Crystallogr. D Biol. Crystallogr..

[36] Morris R, Perrakis A, Lamzin V (2003). ARP/wARP and automatic interpretation of protein electron density maps. Methods Enzymol..

[37] Emsley P, Cowtan K (2004). Coot: Model-building tools for molecular graphics. Acta Crystallogr. D Biol. Crystallogr..

[38] Murshudov G, Vagin A, Dodson E (1997). Refinement of macromolecular structures by the maximum-likelihood method. Acta Crystallogr. D Biol. Crystallogr..

[39] Davis IW (2007). MolProbity: all-atom contacts and structure validation for proteins and nucleic acids. Nucleic Acids Res..

[40] Kleywegt, G. J., Zou, J. Y., Kjeldgaard, M. & Jones, T. A. International tables for crystallography, Vol. F. *Crystallography of Biological Macromolecules* (Rossmann, M. G. and Arnold, E., eds) Chapter 17.1, pp. 353–356, 366–367, (Dordrecht: Kluwer Academic Publishers, Netherlands, 2001).

