# Supplementary material for: Structure and mechanism of a dehydratase/decarboxylase enzyme couple involved in polyketide β-methyl branch incorporation
Source: Sci Rep. 2020 Sep 18;10:15323. doi: 10.1038/s41598-020-71850-w (PMC7501309; doi:10.1038/s41598-020-71850-w)
Supplement: Supplementary file 1 — Supplementary Information 1. [file 41598_2020_71850_MOESM1_ESM.docx]

**Supplementary Information for:**

**Structure and mechanism of a dehydratase/decarboxylase enzyme couple involved in polyketide β-methyl branch incorporation**

Asha V. Nair, Alice Robson, Thomas D. Ackrill, Marisa Till, Matthew J. Byrne, Catherine R. Back, Kavita Tiwari, Jonathan A Davies, Christine L. Willis and Paul R. Race

**Supplementary Figures**

**Figure S1.** Superposition of the active sites of PksI (orange) and CurF (blue). Key active site residues are highlighted in each structure. Secondary structure elements are numbered based on the PksI structure.


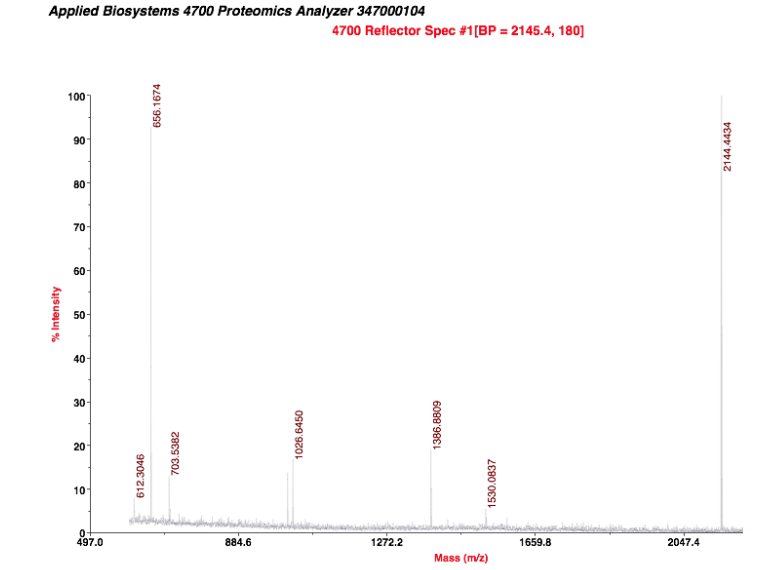


**Figure S2.** MALDI MS analysis of in-gel tryptic digests of co-expressed PksH/PksI following thrombin treatment and HisPur Ni-NTA repurification.

**
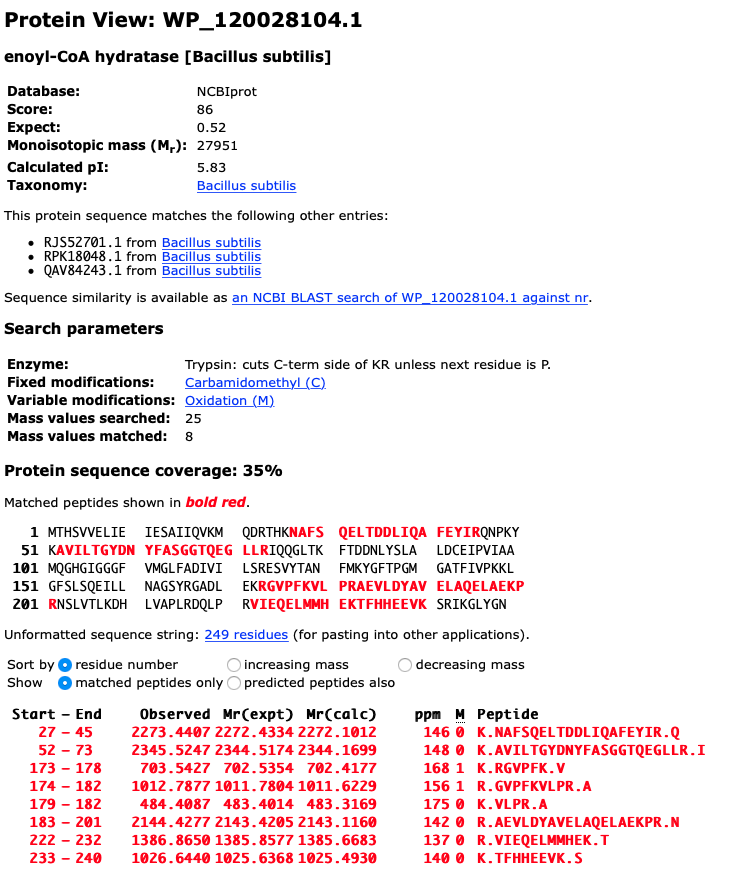
**

**Figure S3.** MASCOT analysis of the data presented in Figure S2.

**Mass spectra of substrates and reaction products:**


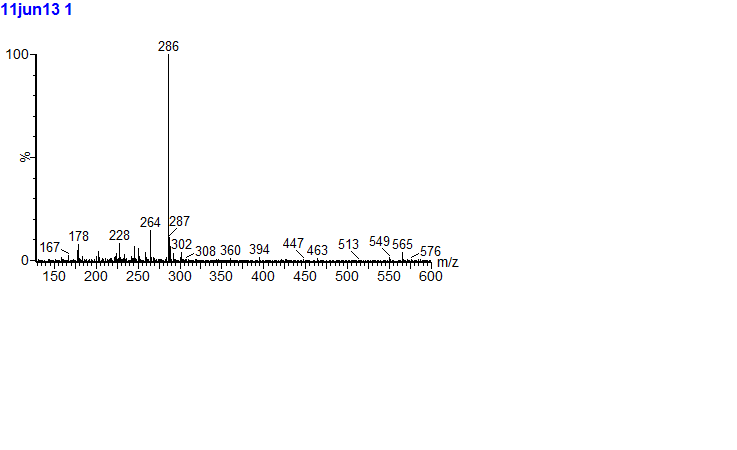


HMG-SNAC (**3**) [M + Na^+^]

synthetic standard


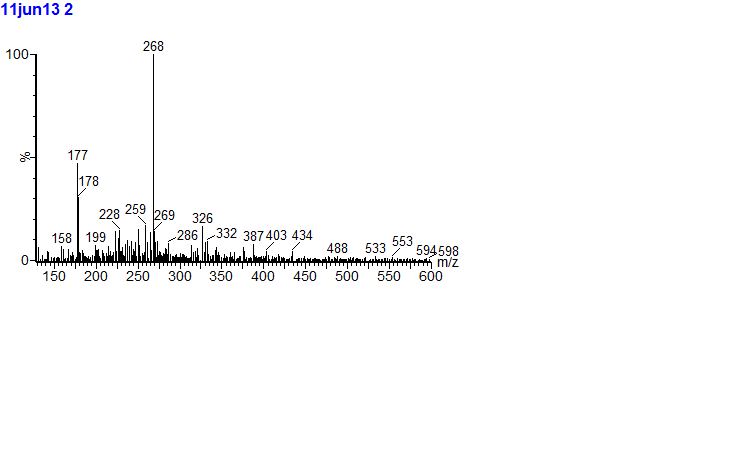


3MG-SNAC (**4**) [M + Na^+^]

PksH reaction product


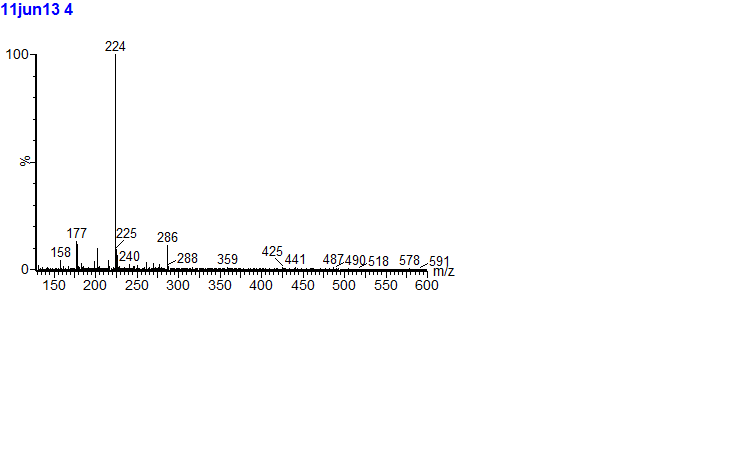


3-MC SNAC (**5**) [M + Na^+^]

PksI reaction product

3-MC SNAC (**5**) [M + H^+^]

synthetic standard


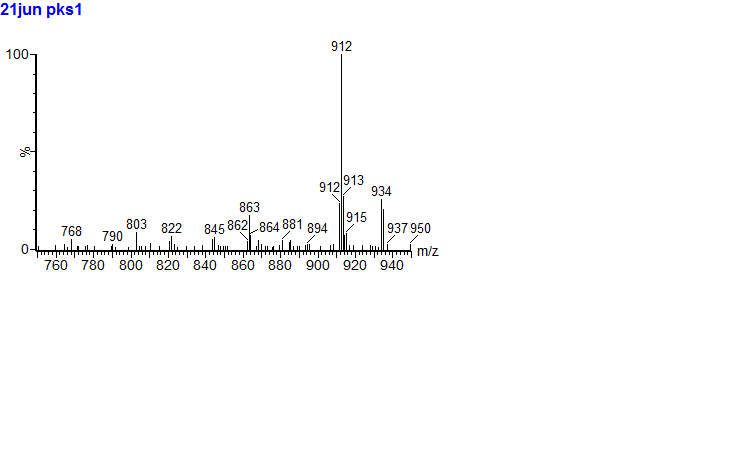


HMG-CoA (**3**) [M + H^+^]

synthetic standard


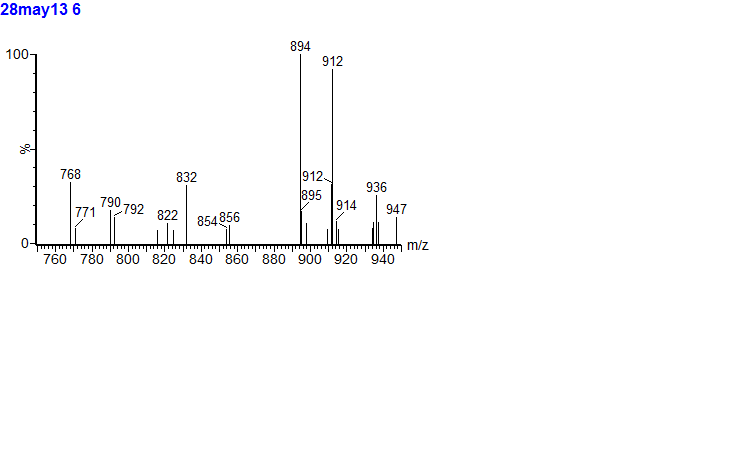


3MG-CoA (**4**) [M + H^+^]

PksH reaction product


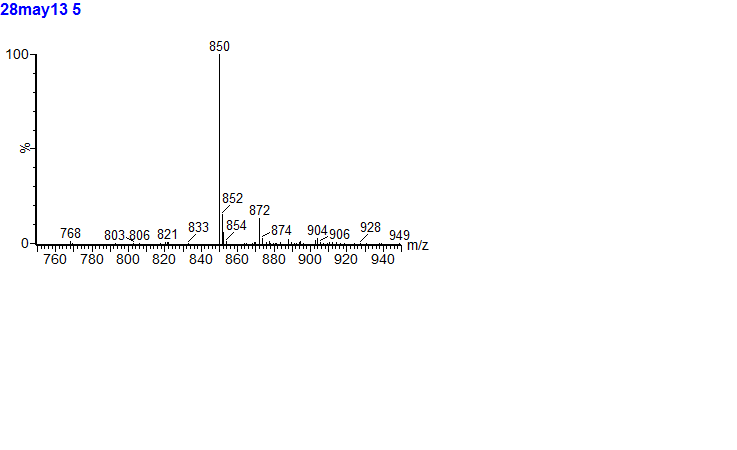


3-MC CoA (**5**) [M + H^+^]

PksI reaction product

**NMR Spectra of substrates and products**

^1^H- NMR (400 MHz, D_2_O)

HMG-SNAC (**3**)

^13^C NMR (100 MHz, D_2_O)

HMG-SNAC (**3**)

^1^H- NMR (400 MHz, D_2_O)

synthetic standard of 3-MC SNAC (**5**)

^13^C NMR (100 MHz, CDCl_3_)

Synthetic standard of 3-MC SNAC (**5**)
